# Range dependent expected utility theory based model for NIMBY conflicts in China: An evolutionary game analysis

**DOI:** 10.1371/journal.pone.0271120

**Published:** 2022-07-12

**Authors:** Hui Zhao, Weihan Wang, Mengran Zhang

**Affiliations:** School of Management Engineering, Qingdao University of Technology, Qingdao, China; Yunnan University of Finance and Economics, CHINA

## Abstract

In recent years, NIMBY(Not In My Backyard) conflicts gradually become hot and difficult in the international community governance, people have realized that the government and people on both sides of the emotional factors have great influences on the results of the conflicts, especially to study the effects of emotion on the evolution of conflicts in China, this article from the following several aspects. First of all, a game model under the influences of emotion is constructed by using Range Dependent Expected Utility(RDEU) theory and emotional function. Secondly, the Jacobian matrix is utilized to analyze the stability of the equilibrium point for the model constructed above. Next, numerical simulation is used to analyze the evolution trend of discrete emotions. The evolutionary results show that when one party holds an optimistic mood, equilibrium evolves to a relatively optimal state; while when one party holds a pessimistic mood, the more pessimistic the party is, the more likely it is to cause NIMBY conflicts. Compared with the people’s sentiments, the government’s moods have a greater impact on the evolutionary consequences. Finally, depending on the conclusions of the evolutionary analysis, some suggestions on the governance of NIMBY conflicts are put forward.

## 1. Introduction

As the growth of the world economy, rapid urbanization process forward, which is associated with a variety of large-scale construction of public facilities. Some of these shared facilities with negative externalities are often conflicted by citizens around the selected site due to their risks or potential risks, causing conflicts and seriously threatening social stability. Such communal facilities are called NIMBY facilities. The NIMBY problem is characterized by contradiction, antagonism, complexity, group evolution and other unconventional characteristics.

In the process of development, different subjects play games around the construction or not and economic disputes [1], the construction of some NIMBY facilities may even cause people’s emotional and psychological aversion, which is called "NIMBY Complex" [2]. With the enhancement of the public’s awareness of community rights and environmental protection, these projects have been criticized and resisted, leading to the increasingly prominent phenomenon of NIMBY conflicts. Starting from developed countries, countries and regions around the world have faced different degrees of NIMBY dilemma. In recent years, problems such as the site selection of Wuhan garbage incineration plant have also attracted widespread attention in China. Depending on the information released by the Ministry of Ecology and Environment, 107 environmental emergencies occurred in the first half of 2020, and the problem of NIMBY conflicts needs to be solved urgently.

Moreover, the problem of NIMBY conflicts is different from the conflict of general construction facilities. With the characteristics of wide influence and strong stimulation, it is easy to cause the government and the public to infect social emotions, forming the phenomenon of group emotional polarization. The instinctive emotions in the group are very easy to infect each other and determine the choice of group behavior [3]. These emotions will change people’s cognition and judgment of the event, leading to the formation of a variety of different conflicts situations. Previous studies have shown that the main reason for NIMBY conflicts is the NIMBY facilities themselves and their siting process [4], mainly reflected in the concern that the projects under construction may affect the quality of life, public health, property values and natural ecosystems in the area [5]. NIMBY conflicts arise when local residents have diverse views on the losses and benefits caused by NIMBY projects [6], and conflicts often arise when stakeholders express distinct priorities for decision-making criteria [7].

Therefore, most scholars consider the hypothesis of economic man. Kunreuther H et al. [8] argued that the pursuit of self-interest maximization is the basic starting point of human behavior, and similarly, the subject of NIMBY conflicts is the same. The higher the amount of monetary compensation, the smaller the possibility of the outbreak of conflict, which has been verified to a certain extent. But there are also research points out that the lucrative compensation is not a cure-all, even some studies pointed out that the monetary compensation for NIMBY facilities be born in essence is a kind of bribery [9]. As a result, the role played by economic factors in the NIMBY conflicts is highly uncertain. After all, human beings are social people and have a mutually beneficial non-self-interested preference in authentic activities [10]. At this time, emotional factors play a strengthening and promoting role in the influence of NIMBY conflicts events, and accelerate the development and evolution process of the event. At the same time, the uncertainty, urgency and severity of NIMBY conflicts will also cause the mood change of the government and affect its cognition and decision-making effect. However, current research on the NIMBY conflicts between the government and the public under the influence of emotion is relatively few, and the theory is not mature enough. Scholars mostly use the stakeholder theory to study the evolution of NIMBY conflicts, such as game theory [11,12], linear and nonlinear integer programming [13], case analysis method [14], and other methods. The above-mentioned literature on NIMBY conflicts effectively relaxed the rational assumptions of participants, explained participants’ behavioral decisions from the perspective of bounded rationality, and lacked the construction of participants’ subjective cognitive level. In particular, it seems difficult to describe irrational behaviors under the influence of emotions. Therefore, from the perspective of emotion, it is of great practical governance significance to study the influence of the emotions of the two main parties in the game on the evolution process of NIMBY conflicts.

On account of this, this paper uses evolutionary game theory, which is distinct from the thought of classical game theory. In the study of NIMBY conflicts, the theory can not only research the contradiction between the government and the public as "economic man", but also analyze the conflicts between the government and the public under the attribute of "social man", so as to provide more scientific suggestions for the governance of NIMBY conflicts in the future. This paper contains the following academic and practical contributions. First of all, this paper constructs the payoff matrix of the government and the public. According to the hierarchical expected utility theory, the utility function is introduced to rank the expected wishes of the two parties, fully considering the subjective wishes of people. The contradiction and conflicts between the government and the public can be better reflected when the replicating dynamic equation is constructed. Secondly, in order to reflect the influence of emotion on the decision-making of both parties in the research, this paper introduces the emotional function when considering the expected benefits of both parties, aiming to reflect the expected benefits of the players in the game process under the effects of different emotions. The first two points are combined to obtain the final replicating dynamic equation of both sides. Thirdly, by setting different emotional values to solve the Nash equilibrium in the corresponding situation, evolution direction of the NIMBY conflicts between the government and the public is analyzed. Finally, based on the solution, MATLAB software is used to conduct numerical simulation analysis on the evolution direction of the conflicts, and the evolutionary trajectory of the NIMBY conflicts can be observed more intuitively.

The rest of this study is structured as follows, Section 2 introduces the research current situation of NIMBY conflicts and emotional impact in NIMBY conflicts. In Section 3, we use evolutionary game theory to construct the payoff matrix based on the government and the public, and according to the Rank Dependent Expected Utility Theory, the emotional function is introduced to construct the replicating dynamic equation. To build the replicating dynamics equation for solving and further simulate emotive impact on the conflicts of the main body role, this paper points four situations for numerical simulation analysis in Section 4. Built on the above analysis, in Section 5, conclusions are drawn and pertinent suggestions are put forward for the management of NIMBY conflicts.

## 2. Literature review

### 2.1 NIMBY conflicts

The emergence of NIMBY incidents is mainly due to the excessive industrial pollution generated by the development of industrialization, and the increasing number of NIMBY incidents has formed an obstacle to the rapid progress of society, so scholars need to take it seriously. Therefore, scholars have carried out systematic theoretical studies on NIMBY conflicts in many aspects, which are mainly divided into three aspects: the notional concept of "NIMBY conflicts", the causes of "NIMBY conflicts" and the governance of "NIMBY conflicts". At the same time, with the deepening of the research, scholars have found that the NIMBY problem involves a wide range of disciplines. Therefore, different scholars conducted studies from different research perspectives, including political science, economics, sociology, communication, psychology and other directions.

In terms of the definition of "NIMBY", O’Hare [15] put forward the concept of "Not On My Block" for the first time in 1977, pointing out that "Not On My Block" was a public facility that brought benefits to the majority of the public, but its external costs were only borne by the residents around the facility, so it was not popular among the surrounding residents and led to protests. British journalist Takahashi LM [16] proposed the concept of NIMBY (Not in My Backyard) in 1980 to describe American protests against chemical waste, then "NIMBY" was used extensively around the world. Since then, scholars have come forward with their own definitions of NIMBY facilities. For instance, Cowan S [17] believed that NIMBY facilities, such as mental health service institutions, had negative externalities and were easily disliked by people, and people were reluctant to stay in them, which was also called "NIMBY syndrome". Teo M et al. [18] believed that NIMBY facilities generally referred to facilities with pollution threat characteristics. After summarizing the predecessors’ definition of NIMBY facilities, as defined by Zhang L et al. [19], NIMBY conflicts can be conceptualized as the resistance and resistance of residents against the construction of NIMBY facilities near their own homes based on the negative effects of NIMBY facilities and unfair cost distribution due to strong self-interest motives and rational trade-off. In terms of the causes of conflicts, most scholars mainly believe that risk perception can better explain the generation of NIMBY effect and the causes of social conflicts, and it has been demonstrated that one of the important reasons for the generation of NIMBY conflicts is the risk perception of residents on NIMBY facilities. For example, Terri M et al. [20] found that citizens blindly followed their NIMBY protests in the NIMBY protests and proposed a "false consensus", which meant that for an NIMBY facility construction project, people believed that the project was of high risk and were more likely to reach a unified false prejudice and such consensus led to the generation of NIMBY conflicts. Starting from the correlation between the public’s risk perception of NIMBY project and behavior choice, Ma L et al. [21] analyzed the factors affecting the public’s risk perception in the construction of nuclear power plants. In other words, citizens have blind conformity to their NIMBY protest behavior in NIMBY protest.

In addition, the differences in risk cognition and interaction barriers between the government and residents may be caused by NIMBY facilities, which also resulted from the different understanding of risk information and risk evidence. For example, regarding the governance of NIMBY conflicts, Devine-Wright P [22] believed that the key to the governance of mass incidents in NIMBY facilities is to change the decision-making mode of "decision-announcement-defense", and advocated the government to build a bottom-up diversified appeal mode of public opinions and a top-down feedback and exchange mechanism, which could taking into account the demands of stakeholders. Depending on the process model of Robert Heath and the action logic theory of participants, Hu XM [23] analyzed the motivation, characteristics and results of stakeholders’ participation in conflicts events in NIMBY facility projects. Madariaga A et al. [24] used static game theory to explain the negative impact of social organizations’ lack of participation in environmental mass incidents on NIMBY problem. Based on the stakeholder theory and through analyzing the strategy selection of the stable evolutionary game for the government, construction enterprises and the surrounding people, Yi G et al. [25] explored the roles and functions played by relevant stakeholders of pollution-related NIMBY facilities in conflicts, and concluded the conditions for promoting the convergence of three-direction evolution and stable strategy points. He XL et al. [26] considered the concerns of stakeholders and established a compensation mechanism for multiple stakeholders in terms of governance body structure and compensation methods. When considering public participation in NIMBY governance, Wang Y et al. [27] believed that the network public opinion played an important role in the establishment of NIMBY facilities, and the smooth development of NIMBY facility PPP project could be promoted from the aspects of constructing multi-agent coordination mechanism and social supervision and security system, attaching importance to public participation and supervision, and designing reasonable incentive scheme. By using Social Cognitive Theory (SCT), Wang Y et al. [28] analyzed the factors affecting public acceptance of NIMBY facilities in Wuhan, and discussed the trade-off relationship between benefits and risks of the community acceptance. He L et al. [29] based on the theory of ground, set up the garbage scene evolution from the crisis factors, using Dynamic Bayesian Network theory to build the dynamic scene of the neighboring avoid waste crisis evolution network, reveals the waste situation evolution from the crisis.

### 2.2 The influence of emotion in NIMBY conflicts

Emotion is an individual’s psychological feeling towards his own state, which is in a state of constant change. Because of this, emotion is also an important factor affecting individual decision-making behavior.However, people are not aware of their own emotional reactions in their daily life, or perception of others’ emotions can help them to obtain more information, so that they can make the optimal choice. Therefore, research on the process of emotional infection has been carried out in the current academic circle. For instance, Elaine H et al. [30] believed that emotional infection referred to the fact that individuals in a group automatically imitated the expressions and actions of others in the group, and gradually achieved emotional convergence by feeling emotional responses. Moreover, emotional infection is a significant link in the interaction between people. When people engage in social communication, emotional convergence effect is often unavoidable. In other words, emotional factors play an important role in the occurrence of collective behavior events, which are unpredictable social interactions that are chaotic and based on the scene at the time. Collective action will occur when a disorganized group of people is stimulated or influenced by some factor to reach a consensus or the consensus on something is destroyed. In the same way, residents living around NIMBY facilities are in the group of "victims", and they can perceive the potential risks of NIMBY facilities. In addition, media reports may continuously expand emotions such as fear, anxiety and anger, which will infect the clusters and play a role of a "booster" for the subsequent protests. For the acts of protest, Hunter S et al. [31] believed that there were numerous influencing factors between the tendency of protest behavior and the implementation of protest behavior. Therefore, the tendency of protest behavior could be measured, and the psychological state of the people could be inferred according to the measured tendency value to predict the occurrence of protest, so as to realize the tendency of protest behavior to replace the actual protest behavior for research.

Correspondingly, Jennifer SL et al. [32] believed that people with psychological emotions such as stress and panic had high-risk cognitive evaluation and could take steps to avoid risks. Moreover, Berkowitz SL [33] confirmed that emotion was an important explanatory variable for people’s protest behavior. In terms of how much trust people have in their government, Jenkins J [34] believed that the public’s distrust of the government has a significant positive impact on the use of violence, demonstrations, marches and other means. However, Joshua KD et al. [35,36] found that the higher the trust of individuals in government institutions, the more likely they were to adopt protest behavior.

At present, more and more scholars have recognized the influence of emotion on NIMBY conflicts, for example, Zhang X et al. [37] considered that anxiety starts from the individual, spreads in the group, and eventually is a kind of psychological state shared by the group. In her research, she believed that social anxiety in the NIMBY incident was one of the important factors that led people to act irrationally. Evensen D et al. [38] believed that a growing public awareness of technology’s potential "side effects" has provided fertile ground for fear and anxiety in the mass NIMBY movement. Dissolving public doubts is the premise to avoid large-scale "NIMBY movement" endangering social stability. Emotion, therefore, plays a critical role in this NIMBY conflicts. This fact provides a viable path to enhance the safety of public policies by strengthening risk communication and improving public participation. Huang C et al. [39–42] believed that the public’s mood would be affected by the public’s risk perception, social trust, conformity psychology, social public opinion and other factors on the NIMBY facilities, thus resulting in cognitive biases that affect the choice of corresponding behaviors. The study on the relationship between biases and behaviors is of profound significance. In addition to the uneven distribution of benefits and risks, from the perspective of motivation, Guo B et al. [43] believed that the effectiveness of emotional release could provide a valid explanation for the spiraling evolution of "Chinese-style" NIMBY conflicts. Therefore, it can be concluded that the prevention and treatment of NIMBY conflicts should not only pay attention to public emotions, but also attach importance to scientific guidance of emotions.

Through comprehensive analysis of the existing literature as you can see, the advancement of urbanization progress prompted a lot of research on NIMBY conflicts, NIMBY conflicts with antagonistic contradictions, complexity, mass evolution these three characteristics, at the same time, the scholars also aware of the emotional contagion is a key factor in the evolution of NIMBY conflicts, from a subjective feeling, attitudes and feelings about the construction of facilities NIMBY mental perception of the NIMBY conflicts, can well predict and explain its behavior decision-making. However, a review of the existing studies shows that: firstly, there are few literatures on the evolution of NIMBY conflicts focusing on the factor of emotion; secondly, the lack of construction on the subjective cognitive level of participants makes it difficult to describe the irrational behaviors under the influence of emotion. Based on this, from the perspective of emotion, this paper uses the theory of Range Dependent Expected Utility(ERDU) to construct the expected benefits of the government and the public in the two big game. Since human beings are bounded rational and constantly learning, therefore, according to the idea of evolutionary game theory, this paper constructs a replicating dynamic equation to further study the evolution of NIMBY conflicts.

## 3. Game theory and model

This section may be divided by subheadings. It should provide a concise and precise description of the experimental results, their interpretation, as well as the experimental conclusions that can be drawn.

### 3.1 Method selection

There are many problems in the decision-making and construction of NIMBY facilities, which are in essence the specific consequences of the game between relevant stakeholders. However, in the preceding analysis, scholars either neglected the dynamic change and learning process when the government and the public game subjects made a strategic choice, or overlooked the emotion as an important influencing factor. Classical game theory generally assumes that the subject is completely rational and can grasp all the pertinent information of the game system, and on this basis, it chooses the strategy with the greatest benefits to execute. All the strategic choices it makes in the environment system are based on the consideration of maximizing its own interests.

The game environment in reality is complex, by contrast, there have many uncertainties in reality, and the influencing factors of the game-agent are also disparate. The existence of these circumstances makes it difficult for the game-agent to be absolutely rational, and the consideration basis for strategic choice is not completely extensive and thorough. However, from the perspective of bounded rationality, evolutionary game theory holds that the information mastered by the participants is incomplete and the strategic choice of the participants is learning and evolving, which is in accordance with the background of the NIMBY conflicts. Therefore, it is more scientific to use the idea of evolutionary game theory to evolve the evolution of government and public behavior strategies. In addition, Gong RT [44] used the rank-based expected utility model to study the equilibrium solution analysis of the classic hawk-dove game when the participants had emotions, and proved that the finding of emotional factors would lead to the non-existence of mixed Nash equilibrium in the hawk-dove game, which solved the doubt that the traditional game analysis could not give a positive answer. Xiong GQ et al. [45] found that participants in group events tended to make "concession" behavior when they were in an "optimistic" mood, while they tended to make "confrontation" behavior when they were in a "pessimistic" mood. All the above analyses take into account the game model of decision-makers with emotions and get the results closer to the real situation. As a consequence, this paper tries to introduce three states of optimism, pessimism and rational emotion into the evolutionary game between the government and the surrounding masses, and analyzes the influence of emotion change on the equilibrium of the evolutionary game between the two sides, so as to provide decision-making reference for the government to establish the emotional supervision and guidance mechanism. It is not only helpful to promote and improve the application of evolutionary game theory in the field of NIMBY facilities to a certain extent, but also to provide a certain scientific basis for government decision-making.

### 3.2 The basic theory

NIMBY conflicts are different from the collision of conventional construction facilities. With the characteristics of wide impact range and strong stimulation, the government and the public are likely to be infected with social emotions, resulting in the polarization of group emotions, and even lead to violent conflicts and a lose-lose situation [46]. Emotional factors play a strengthening and promoting role in the influence of NIMBY conflicts events and accelerate the development and evolution process of the events [47]. At the same time, the uncertainty, urgency and severity of NIMBY conflicts will also cause the mood change of the government and affect its cognition and decision-making effect. Therefore, the government and the public, as the core subjects of conflicts, constantly adjust their own attitudes and behaviors, contributing to diverse trends of conflicts evolution. In this paper, the government and the people as the research object, according to the RDEU theory, introducing the utility function and emotional function, evolutionary game method and combining the building NIMBY conflicts in the evolution process of the government and people strategies of dynamic game model, analyzing the government and the people’s emotion factors influence on NIMBY collisions evolution, aiming to provide a more scientific and referential suggestions for the current environment of adjacent governance.

RDEU theory is a utility theory that includes individual psychological preferences and emotions. This theory takes humanity’s incomplete rationality into account. Its core is a real valued function *V*(*x*) defined by the comprehensive utility function *U*(*x*) and decision weight function *π*(*x*) to represent individual’s preference for a strategy [48], that is: $V(x_{i})=\pi (x_{i})*U(x_{i})$, where, for strategy set *x* = {*x*_*i*_; *i* = 1,2,…*n*}, the probability that *x* takes *x*_*i*_ is *P*{*X* = *x*_*i*_} = *P*_*i*_. Sort the strategy *x*_*i*_ by *U*(*x*) and specify *x*_1_>*x*_2_>⋯>*x*_*n*_. In this case, the utility level of *x*_*i*_ is defined as *R*_*i*_, and the strategy of the probability distribution function of $R_{i}=P\{X\leq x_{i}\}=P_{i}+P_{\left(i+1\right)}$. At this point, $\pi (x_{i})=w(1+P_{i}-R_{i})-w(1-R_{i})$. Among them, *w*(∙) is a monotonically increasing emotional function, which is subject to *w*(0) = 0 and *w*(1) = 1 [49]. In other words, RDEU theory integrates the hierarchical distribution function and the emotional function to obtain the cumulative nonlinear decision weight, which can describe the emotional state and the influence of emotional intensity of the government and the public under the condition of uncertainty.

Evolutionary game theory is an application of the mathematical framework [50] to the dynamics of animal conflicts (including, of course, conflicts people engage in). In game theory, the object is to find an appropriate strategy to resolve arising conflicts, or alternatively to find the optimal sequence of decision that leads to the highest payoff. Maynard coined the term "Evolutionary Stable Strategy" (ESS), to mean a move (or play) that would assure the type of animal that wields it an evolutionary (that is, Darwinian) advantage over the opponent, in the sense that the ESS could never become extinct (but it may have to coexist with other strategies that are also ESS). The concept of the ESS is related to the Nash equilibrium (the rational choice of strategy in economic games), but it is more refined: while every ESS is a Nash equilibrium, some Nash equilibria are not ESS because they are unstable fixed points of the evolutionary dynamics [51–53]. Moreover, Weibull introduced evolutionary stability and replicating dynamics [54] where ideas from evolutionary biology and rationalistic economics meet emphasizing the links between static and dynamic approaches and noncooperative game theory. The essence of the replicating dynamic equation is a differential equation, which is used to characterize the rate at which the proportion of individuals adopting a certain strategy in the total population changes over time, which is mainly related to the fitness of the current individuals and the proportion of individuals in the population. If *x* = {*i*, *j*} is the pure strategy set of the main body of the evolutionary game, at time *t*, when the game subject and other players both choose strategy *i* or strategy *j*, the fitness of the game subject is *U*_*t*_(*i*, *i*) or *U*_*t*_(*j*, *j*); when other players choose strategy i but the game subject chooses strategy j, the fitness is *U*_*t*_(*j*, *i*); otherwise, it is *U*_*t*_(*i*, *j*). At the same time, *S*_*t*_(*i*) is the proportion of game players choosing strategy *i* in the group, and the ratio of the game subject choosing strategy *j* in the group is 1−*S*_*t*_(*i*). Then, the replicating dynamic equation of the subject of strategy *i* is:

$$F(s)=s_{t}\prime (i)=s_{t}(i)(U_{t}(i)-U_{t})$$

Based on the evolutionary game theory, the mechanism and principle of the replicating dynamic equation predicting the evolution of collective behavior in a group can be explained: assuming that strategy *i* is the strategic selection of a small mutant group and strategy *j* is the strategic selection of a large group, it is not difficult to see from the replicating dynamic equation that when the fitness of small mutant group is high, *F*(*s*)>0, the strategic selection of the population will converge to the strategic selection of the individuals of the small mutant population. When the fitness of the small mutant population is low, *F*(*s*)<0, the strategic selection of the group will converge to the strategic selection of the large group. The evolution and change of collective behavior can be attributed to analyse the convergence process of group strategy.

### 3.3 The basic assumptions

Hypothesis 1: In the absence of other constraints, since neither the government nor the public have perfect information at the initial stage of the construction of NIMBY facilities, the government will make two decisions: negotiation and closure; while the public will understand or resist the construction of NIMBY facilities in their "own backyard". The selection of consultative decision-making by the government is mainly derived from the idea of "NIMBY management and control". It is believed that the government, as a "visible hand", should fulfill its regulatory responsibilities. In fact, the choice of consultative strategy means that the government adopts a more active supervision mode, allowing the public to participate in decision-making with reservation and releasing information to the surrounding masses selectively, which can relatively reduce social panic and unrest on the basis of protecting the public’s right to know and supervise. Adoption of closure decision means that the government chooses a passive supervision mode and completely locks up the information. On the one hand, it can reduce and avoid the possibility of direct conflicts with the surrounding people, because there are not many people who know the information in the initial stage, which is conducive to control. On the other hand, an agreement can be reached with the construction enterprise that is more beneficial to both sides. Assuming that the probability of the government taking a compromise decision is *p*, then the probability of the government taking a closed decision is 1−*p*, where 0≤*p*≤1. At the same time, in the face of the establishment of NIMBY facilities, the public may be willing to sacrifice part of their own interests in consideration of national and social interests, so as to take an understanding decision and agree to the government to build NIMBY facilities near their residence. On the contrary, if the people are not willing to sacrifice the ego for the greater ego, they will adopt protest tactics such as appeals, demonstrations and violent conflicts. Assuming that the probability of the public taking the understanding strategy is *q*, then the probability of taking the resistant decision is 1−*q*, where *q*∈[0, 1].

Hypothesis 2: Assume that under a different combination of strategies, the payoff matrix of the government and the public are presented in Table 1. At this point, it is especially important to measure how much the government and the public gain from a combination of different strategies. On the one hand, for the government, when people choose to the strategy of understanding, the government will bring benefits as long as it can successfully establish NIMBY facilities, no matter whether it adopts a strategy of negotiation or closure. However, when the government adopts the strategy of negotiation, it will gain additional strong credibility by increasing its prestige in the eyes of the people, therefore *α*>*γ*. On the other hand, considering that the degree of public conflict largely depends on whether the government chooses to the strategy of negotiation or closure, so the analysis is carried out on two aspects. Under the decision of closure, the number of people rebelling is relatively small to some extent because they are not informed in advance, which leads to fewer conflicts. In contrast, the government’s choice of negotiation may increase the likelihood of widespread resistance, and the consequences may be more serious if the government does not have sufficient capacity to appease the masses. Hence, the government will spend less on the decision of closure, so *δ*>*β*. In the similar way, for the public, regardless of whether the government adopts the strategy of negotiation or closure, the public will receive compensatory benefits from the government if they adopt the decision of understanding and the amount of compensation received depends on which decision the government takes. Obviously, when the government makes a closed decision, it will have a kind of "guilt" mentality, so it will want to use more economic benefits to calm down the crowd, so that the public will get more economic benefits than the government when it makes a decision of negotiation, namely *η*>*ε*. In addition, when the public takes the decision to resist, it is similar to the analysis of the previous part of the government, a closed approach by the government would reduce the number of civil conflicts, so *θ*>*ζ*. Combined with the above analysis, it can conclude that *α*>*γ*>*δ*>*β* and *η*>*ε*>*θ*>*ζ*.

**Table 1:** Payoff matrix of government and public.

**Public** **Government**	**Understanding (*q*)**	**Resistance (1−*q*)**
**Negotiation(*p*)**	(*α*, *ε*)	(*β*, *ζ*)
**Closure(1−*p*)**	(*γ*, *η*)	(*δ*, *θ*)

Hypothesis 3: In the game process, the emotional function of both sides is introduced as *w*(*p*) = *p*^*r*1^ and *w*(*q*) = *q*^*r*2^, where *r*_1_ and *r*_2_ represent the sentiment index of the government and the public respectively, and *r*_1_, *r*_2_>0. In particular, when *r*_1_ = 1 and *r*_2_ = 1, it means that both the government and the public are not subject to emotions, that is to say, both sides maintain absolute rationality.

### 3.4 Construct an evolutionary game model based on RDEU theory

According to the above analysis and assumptions, this paper establishes the game payoff matrix between the government and the public. According to the RDEU theory’s definition of hierarchy and decision-making weight and the hypothetical conditions of income, the strategic payoff, strategic probability, and utility level and weight of both the government and the public can be obtained, as shown in Tables 2 and 3. From Tables 2 and 3, the expected utility and its average expected utility of the government and the public when they choose different strategies can be obtained. Suppose the utility of the government adopting the negotiation strategy is *U*_*P*_, and its expected utility level is denoted as *E*(*U*_*p*_), while the utility of the public selecting the understanding strategy is *U*_*q*_, and its expected utility is regarded as *E*(*U*_*q*_). Then, based on RDEU theory, the utility and expected utility levels of the government are:

$$U_{p}=\alpha *q^{r2}+\beta *(1-q^{r2})=\beta +(\alpha -\beta )*q^{r2}$$ (1)

$$\begin{array}{l} E(U_{p})=\alpha *w(pq)+\gamma *[w(q)-w(pq)]+\delta *[w(1-p+pq)-w(q)]+\beta *[1-w(1-p+pq)]\\ \;=(\alpha -\gamma )*{\left(pq\right)}^{r1}+(\gamma -\delta )*q^{r1}+(\delta -\beta )*{\left(1-p+pq\right)}^{r1}+\beta \end{array}$$ (2)

**Table 2:** Revenue, probability, rank and weight of the government.

Revenue	Probability	Rank	Weight
*α*	*pq*	1	*w*(*pq*)
*γ*	(1−*p*)*q*	*pq*	*w*(*q*)−*w*(*pq*)
*δ*	(1−*p*)(1−*q*)	1−*q*	*w*(1−*p*+*pq*)−*w*(*q*)
*β*	*p*(1−*q*)	*p*(1−*q*)	1−*w*(1−*p*+*pq*)

**Table 3:** Income, probability, grade and weight of the people.

Income	Probability	Rank	Weight
*ϵ*	(1−*p*)*q*	1	*w*(*q*−*pq*)
*ζ*	*pq*	*q*+*pq*	*w*(*q*)−*w*(*q*−*pq*)
*η*	(1−*p*)(1−*q*)	1−*q*	*w*(1−*p*+*pq*)−*w*(*q*)
*θ*	*p*(1−*q*)	*p*(1−*q*)	1−*w*(1−*p*+*pq*)

Similarly, it can be concluded that the utility and expected utility levels of the public are:

$$U_{q}=\varepsilon *p^{r1}+\eta *(1-p^{r1})=\eta +(\varepsilon -\eta )*p^{r1}$$ (3)

$$\begin{array}{l} E(U_{q})=\eta *w(q-pq)+\varepsilon *[w(q)-w(q-pq)]+\theta *[w(1-p+pq)-w(q)]+\zeta *[1-w(1-p+pq)]\\ \;=(\eta -\varepsilon )*{\left(q-pq\right)}^{r2}+(\varepsilon -\theta )*q^{r2}+(\theta -\zeta )*{\left(1-p+pq\right)}^{r2}+\zeta \end{array}$$ (4)

On this basis, considering that the government and the public meet the conditions of bounded rationality and have the characteristics of learning, imitation and adjustment strategies, etc.. Therefore, through the replicating dynamic equation can describe the mood in the evolution game theory under the influence of the strategy of learning, imitation and final stable evolution process, further build based on the RDEU theory of evolutionary game model to avoid conflicts, in order to analyze the government and the public mood influence the result of the game. The replicating dynamic equation is constructed as following:

$$\begin{array}{l} \frac{\partial p}{\partial t}=p^{r1}[Up-E(Up)]\\ \;=p^{r1}[(\alpha -\beta )q^{r2}-(\alpha -\gamma ){\left(pq\right)}^{r1}-(\gamma -\delta )q^{r1}-(\delta -\beta ){\left(1-p+pq\right)}^{r1} \end{array}$$ (5)

$$\begin{array}{l} \frac{\partial q}{\partial t}=q^{r2}[Uq-E(Uq)]\\ \;=q^{r2}[(\eta -\zeta )+(\varepsilon -\eta )p^{r1}-(\eta -\varepsilon ){\left(q-pq\right)}^{r2}-(\varepsilon -\theta )q^{r2}-(\theta -\eta ){\left(1-p+pq\right)}^{r2} \end{array}$$ (6)

The Nash equilibria and the evolutionary stable strategies of the game can be solved according to its utility function and the replicating dynamic equation.

## 4. Model solution and numerical simulation analysis

**Fig 1:**
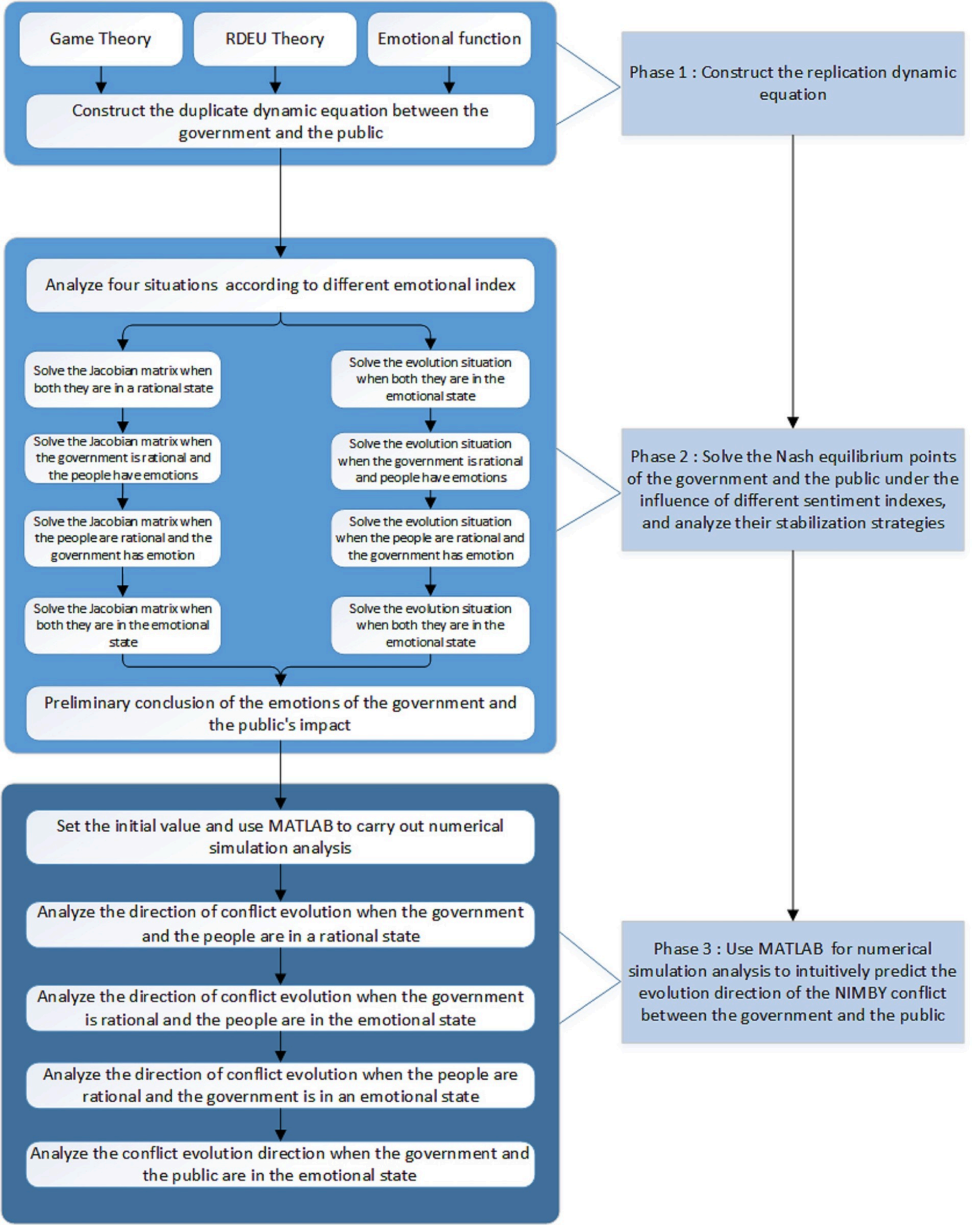
The logical framework. The right column of the figure is three stages analyzed in this paper, and the left column of the figure is the specific theories and methods used in the current stage.

In this section, the Rank Dependent Expected Utility theory and numerical simulation method are proposed, consisting of these three stages. Firstly, RDEU theory is used to construct the replicating dynamic equation of the government and the public (shown in Section 3) in combination with the emotive function. Then, according to the Jacobian matrix of the government and the public, the Nash equilibrium points of the two parties under the influence of unusual emotion indexes are solved, and the stability strategies of the two parties are analyzed. Finally, MATLAB software is utilized for numerical simulation analysis to more intuitively predict the evolution direction of the NIMBY conflicts between the government and the public. Accordingly, Fig 1 illustrates the logical framework of this paper.

### 4.1 Solving and analysis of the game model

The game adopts a mixed strategy, and the Nash equilibrium point and its existence can be solved through its utility function, and let:

$$\left\{ \begin{array}{l} Vp=Up-E(Up)=0\\ Vq=Uq-E(Uq)=0 \end{array}\right.$$ (7)

Nash equilibrium point can be obtained from the transcendental Eq (7), denoted as (p*, q*), then five stable equilibrium points of the evolution of the game system are obtained: *E*_1_(0, 0), *E*_2_(0, 1), *E*_3_(1, 0), *E*_4_(1, 1) and *E*_5_(p*, q*). According to the evolutionary game theory, *Det*(*J*)>0 and *Tr*(*J*)<0, is the condition to judge the stability of the five evolutionary equilibrium points, and determine the final evolutionary stable strategy of the game under the influence of emotion. Let:

$$\left\{ \begin{array}{l} F(p)=\partial p/\partial t\\ F(q)=\partial q/\partial t \end{array}\right.$$ (8)

Take the partial derivatives of Eq (8) with respect to *p* and *q*, respectively, to obtain Eq (9):

$$\frac{\partial F(p)}{\partial p}=r_{1}p^{r1-1}[(\alpha -\beta )q^{r2}-(\alpha -\gamma ){\left(pq\right)}^{r1}-(\gamma -\delta )q^{r1}-(\delta -\beta ){\left(1-p+pq\right)}^{r1}]+r_{1}p^{r1}[(\gamma -\alpha )p^{r1-1}q^{r1}-(q-1)(\delta -\beta ){\left(1-p+pq\right)}^{r1-1}]$$

$$\frac{\partial F(p)}{\partial q}=p^{r1}[r_{2}(\alpha -\beta )q^{r2-1}-r_{1}(\alpha -\gamma )p^{r1}q^{r1-1}-r_{1}(\gamma -\beta )q^{r1-1}-r_{1}p(\delta -\beta ){\left(1-p+pq\right)}^{r1-1}]$$

$$\frac{\partial F(q)}{\partial p}=q^{r2}[r_{1}(\varepsilon -\eta )p^{r1-1}+r_{2}q(\eta -\varepsilon ){\left(q-pq\right)}^{r2-1}-r_{2}(q-1)(\theta -\zeta ){\left(1-p+pq\right)}^{r2-1}]$$

$$\frac{\partial F(q)}{\partial q}=r_{2}p^{r2}[(p-1)(\eta -\varepsilon ){\left(q-pq\right)}^{r2-1}-(\varepsilon -\theta )q^{r2-1}-p(\theta -\zeta ){\left(1-p+pq\right)}^{r2-1}]+r_{2}q^{r2-1}[(\eta -\zeta )+(\varepsilon -\eta )p^{r1}-(\eta -\varepsilon ){\left(q-pq\right)}^{r2}-(\varepsilon -\theta )q^{r2}-(\theta -\zeta ){\left(1-p+pq\right)}^{r2}]$$ (9)

It can be found that the equilibrium of the game and the evolutionary stability of each point depend on the income parameter and the emotional index in the model. According to the theory of Rank Dependent Expected Utility, this paper divides the possible emotional states of the government and the public into three dimensions: positive, rational and negative, and takes optimistic (*r*>1), rational (*r* = 1) and pessimistic (*r*<1) to describe the emotional state of both parties. The value of the emotional index *r* represents the intensity of emotion.

Considering the different situations of NIMBY conflicts, we will discuss the situation when the government and the public hold different emotions. There are four situations:

(1) Both the government and the public are in a rational state
  This state means that both the government and the public are not affected by emotions, that is, *r*_1_ = *r*_2_ = 1. At this point, the Nash equilibrium point can be obtained by substituting the emotional function into Eq (7), the results are p* = (*η*−*θ*)/(*η*+*ζ*−*θ*−*ε*) and q* = (*β*−*δ*)/(*β*+*γ*−*α*−*δ*). When both sides are without emotional state, therefore, the government chooses the negotiation strategy with the probability of (*η*−*θ*)/(*η*+*ζ*−*θ*−*ε*), and the public chooses the understanding strategy with the probability of (*β*−*δ*)/(*β*+*γ*−*α*−*δ*). Furthermore, the parameters of the Jacobian matrix are obtained by substituting each parameter into Eq (9), as showed in Table 4.

**Table 4:** The Jacobian matrix parameter value under the rational state of the both parties.

Equilibrium point	∂*F*(*p*)/∂*p*	∂*F*(*p*)/∂*q*	∂*F*(*q*)/∂*p*	∂*F*(*q*)/∂*q*
*E*_1_(0, 0)	*β*−*δ*	*0*	*0*	*η*−*θ*
*E*_2_(0, 1)	*α*−*γ*	*0*	*0*	*θ*−*η*
*E*_3_(1, 0)	*δ*−*β*	*0*	*0*	*ε*−*ζ*
*E*_4_(1, 1)	*γ*−*α*	*0*	*0*	*ζ*−*ε*
*E*_5_(p*, q*)	*0*	(*α*+*δ*−*β*−*γ*)(*p*−*p*^2^)	(*η*+*ζ*−*θ*−*ε*)(*q*^2^−*q*)	*0*

According to the calculated Jacobian matrix parameters, *Det*(*J*) and *Tr*(*J*) can be obtained, and Table 5 includes the stability of the equilibrium points. As showed in Table 5, when both sides of the game are rational, the determinants and trails of the Jacobian matrix corresponding to points *E*_1_, *E*_2_, *E*_3_ and *E*_4_ are uncertain, so they do not reach a stable state. For the mixed strategy Nash equilibrium point *E*_5_, when *η*+*ζ*<0, on account of its *Det*(*J*)>0 and *Tr*(*J*) = 0, this point is tending to be stable at this time, and unstable on the contrary. Hence, it is stable in one direction and unstable in another, which can be viewed as a saddle point. In other words, both sides do not choose a pure strategy explicitly in a rational state, but constantly adjust their own strategies according to the situation. The above result is discussed under the ideal assumption of the rational state. Nevertheless, in reality, it is difficult to see the situation that both the government and the people are in a rational state.

**Table 5:** The evolution in which both the government and the public are in a rational state.

Equilibrium point	*Det*(*J*)	*Tr*(*J*)	Stability
*E*_1_(0, 0)	−	uncertainty	instability
*E*_2_(0, 1)	−	uncertainty	instability
*E*_3_(1, 0)	+	+	instability
*E*_4_(1, 1)	+	−	instability
*E*_5_(p*, q*)	+/−	0	saddle point

When *Det*(*J*)>0∩*Tr*(*J*)<0, the equilibrium point is an ESS.

(2) The government is rational and the people are in an emotional state

Under this situation, the government in a rational state and people hold emotions, which means that the people in behavioral decision to the intervention by the mood, because public access to information is not complete, understanding of events of subjectivity is strong, so this kind of situation is common in NIMBY conflicts.

In this scenario, $r_{2}\neq 1,\;r_{1}=1,\;{p^{*}=p}_{r2}^{*},\;q^{*}=q_{r2}^{*},\;(p_{r2}^{*},\;q_{r2}^{*})$ are the solutions of the transcendental Eq (7). The parameters are substituted into Eq (9) to calculate the parameters of the Jacobian matrix and the evolutionary stability of each point, as showed in Tables 6 and 7.

**Table 6:** Jacobian matrix parameter values under rational government and emotional public.

Equilibrium point	∂*F*(*p*)/∂*p*	∂*F*(*p*)/∂*q*	∂*F*(*q*)/∂*p*	∂*F*(*q*)/∂*q*
*E*_1_(0, 0)	*β*−*δ*	*0*	*0*	*0*
*E*_2_(0, 1)	*α*−*γ*	*0*	*0*	*r*_2_(*θ*−*η*)
*E*_3_(1, 0)	*δ*−*β*	*β*−*α*	*0*	*0*
*E*_4_(1, 1)	*γ*−*α*	(*r*_2_−1)(*α*−*β*)	(*r*_2_−1)(*η*−*ε*)	*r*_2_(*ζ*−*ε*)
*E*_5_(p*, q*)	Nash equilibrium depends on incomes and sentiment index			

**Table 7:** The evolution of rational government and the emotional state of the public.

Equilibrium point	*Det*(*J*)	*Tr*(*J*)	Stability
*E*_1_(0, 0)	0	−	instability
*E*_2_(0, 1)	−	uncertainty	instability
*E*_3_(1, 0)	0	+	instability
*E*_4_(1, 1)	+/−	uncertainty	instability
*E*_5_(p*, q*)	Stability depends on incomes and sentiment index		

When *Det*(*J*)>0∩*Tr*(*J*)<0, the equilibrium point is an ESS.

According to the results in Table 7, when the government holds the emotional state, the equilibrium point *E*_1_ corresponds to *Tr*(*J*)<0, but the *Det*(*J*) = 0, so it does not meet the evolutionary stability condition, and the determinant of Jacobian matrix corresponding to *E*_4_ exists greater than 0, but its trail is uncertain, so neither is an evolutionary stable strategy. Therefore, it can be observed that the government is rational and the public does not tend to adopt pure strategies when holding emotions. At the Nash equilibrium point of mixed strategies, *E*_5_, its stability is difficult to judge because its sentiment index *r*_2_ and incomes are uncertain, and different values of variables will produce different results.

(3) The people are rational and the government is in an emotional state

In this case, $p^{*}=p_{r1}^{*},\;q^{*}=q_{r1}^{*}$, the Nash equilibrium point of mixed strategy is $\left(p_{r1}^{*},\;q_{r1}^{*}\right)$. The parameters are substituted into Eq (9) to calculate the parameters of the Jacobian matrix and the evolutionary stability of each point, as showed in Tables 8 and 9.

**Table 8:** Jacobian matrix parameter values under emotional government and rational public.

Equilibrium point	∂*F*(*p*)/∂*p*	∂*F*(*p*)/∂*q*	∂*F*(*q*)/∂*p*	∂*F*(*q*)/∂*q*
*E*_1_(0, 0)	*0*	*0*	*0*	*η*−*θ*
*E*_2_(0, 1)	*0*	*0*	*η*−*ε*	*θ*−*η*
*E*_3_(1, 0)	*0*	*α*−*β*	*0*	ε−ζ
*E*_4_(1, 1)	*r*_1_(*γ*−*α*)	(1−*r*_1_)(*α*−*β*)	*0*	*ζ*−*ε*
*E*_5_(p*, q*)	Nash equilibrium depends on incomes and sentiment index			

**Table 9:** The evolution of rational people and emotional state of government.

Equilibrium point	*Det*(*J*)	*Tr*(*J*)	Stability
*E*_1_(0, 0)	0	0	saddle point
*E*_2_(0, 1)	0	+	instability
*E*_3_(1, 0)	0	+	instability
*E*_4_(1, 1)	+	−	ESS
*E*_5_(p*, q*)	Stability depends on incomes and sentiment index		

When *Det*(*J*)>0∩*Tr*(*J*)<0, the equilibrium point is an ESS.

When the government holds the emotional state, Table 9 lists that the equilibrium points *E*_2_ and *E*_3_ do not satisfy the *Det*(*J*)>0 and *Tr*(*J*)<0. However, when *η* = *θ*, the partial derivatives of *E*_1_ are all 0, so *E*_1_ belongs to the saddle point. Therefore, when the public is rational and the government holds emotions, it tends to adopt (negotiate, understand) pure strategies. For Nash equilibrium point *E*_5_ of mixed strategies, due to the influence of sentiment index *r*_1_ and the uncertainty of profit value, its stability is difficult to judge, and different values of variables will produce different results.

(4) Both the government and the public are in an emotional state

In this scenario, both government and people are in the emotional state of mind, which is the combination of optimistic and pessimistic mood, this emotional state can lead to both sides of the cognitive and judgment in decision-making deviation, thus influence each other possible strategy. At this point, *r*_1_≠1, *r*_2_≠1. If *p* = p* and *q* = q* exist, so that Eq (7) holds, it indicates that a new mixed strategy Nash equilibrium appears under the influence of the emotions of both parties. Although the above transcendental equations are difficult to solve, it is not hard to prove that there exists some $q=q_{\left(r_{1},p\right)}^{*}$ such that *V*_*P*_ = 0, and similarly, there exists some $p=p_{\left(r_{2},q\right)}^{*}$ that satisfies *V*_*q*_ = 0. In addition, when the emotions of both parties are in an extreme state, that is *r*_1_→0 and *r*_2_→0, or *r*_1_→±∞ and *r*_2_→±∞, the Nash equilibrium does not exist. Therefore, when considering the emotional factors of both sides, the probability that the government chooses the negotiation strategy in Nash equilibrium is $p_{\left(r_{2},q\right)}^{*}$, and the probability that the public chooses the understanding strategy is $q_{\left(r_{1},p\right)}^{*}$, then the Jacobian matrix parameters and evolutionary stability of each point are shown in Tables 10 and 11.

**Table 10:** Jacobian matrix parameter values under both the government and the public have emotional states.

Equilibrium point	∂*F*(*p*)/∂*p*	∂*F*(*p*)/∂*q*	∂*F*(*q*)/∂*p*	∂*F*(*q*)/∂*q*
*E*_1_(0, 0)	*0*	*0*	*0*	*0*
*E*_2_(0, 1)	*0*	*0*	*r*_2_(*η*−*ε*)	*r*_2_(*θ*−*η*)
*E*_3_(1, 0)	*0*	*0*	*0*	ε−ζ
*E*_4_(1, 1)	*r*_1_(*γ*−*α*)	(*r*_2_−*r*_1_)(*α*−*β*)	*r*_2_(*η*−*ε*)	*r*_2_(*ζ*−*ε*)
*E*_5_(p*, q*)	Nash equilibrium depends on incomes and sentiment index			

**Table 11:** The evolution of both the government and the public with emotional states.

Equilibrium point	*Det*(*J*)	*Tr*(*J*)	Stability
*E*_1_(0, 0)	0	0	saddle point
*E*_2_(0, 1)	0	−	instability
*E*_3_(1, 0)	0	0	saddle point
*E*_4_(1, 1)	+/−	−	ESS/NOT
*E*_5_(p*, q*)	Stability depends on incomes and sentiment index		

When *Det*(*J*)>0∩*Tr*(*J*)<0, the equilibrium point is an ESS.

As shown in Table 11, when both sides hold emotions, it can be found that due to the mutual influence of emotions of both sides, it is difficult for the government and the public to estimate the behavior of the other side and make a favorable choice. However, no matter what kind of emotions the two sides hold, if the government has the exact same type of emotions more strongly, namely *r*_1_>*r*_2_, the game will appear evolutionary stable strategy (negotiation, understanding). Although *E*_1_ and *E*_3_ do not meet the stability conditions, their partial derivatives are all 0 and they are both saddle points, which means that for the Nash equilibrium point *E*_5_ of the mixed strategy, under the interaction of emotions of both sides, the mixed strategy may turn into a pure strategy, and then become an evolutionary stable strategy.

Based on the above analysis, it can be observed that in the situation where one or both parties hold emotions, compared with the rational state, the evolutionary system of behavioral decision has obvious changes. On the one hand, when both parties adopt a mixed strategy, the probability of Nash equilibrium point *E*_5_ and its evolutionary stability will be modified by the influence of the party with emotion, and may even become a pure strategic Nash equilibrium. On the other hand, under different emotional combinations, emotion can make pure strategy become revolutionaries stable strategy, which has different results from a rational state game.

### 4.2 Numerical simulation analysis

The foregoing analysis shows that the two sides held in different emotions to the evolution of the NIMBY conflicts has very important influence, due to the evolution of each point is also depends on the size of the profit and emotional strength, especially the evolution of the Nash equilibrium stability, and this kind of data is difficult to get from the event. In this paper, Matlab is used to carry out numerical simulation analysis on the evolutionary game. Under the premise of satisfying Hypothesis 2, the parameters are reduced to *α* = 15, *β* = −2, *γ* = 7, *δ* = 4, *ε* = −4, *ζ* = −4, *η* = 10, *θ* = −1 for the convenience of analysis. And set *p* = 0.5 and *q* = 0.5 as the initial probability for simulation, to analyze the evolution trajectory of different emotional combinations and different emotional indexes.

**Fig 2:**
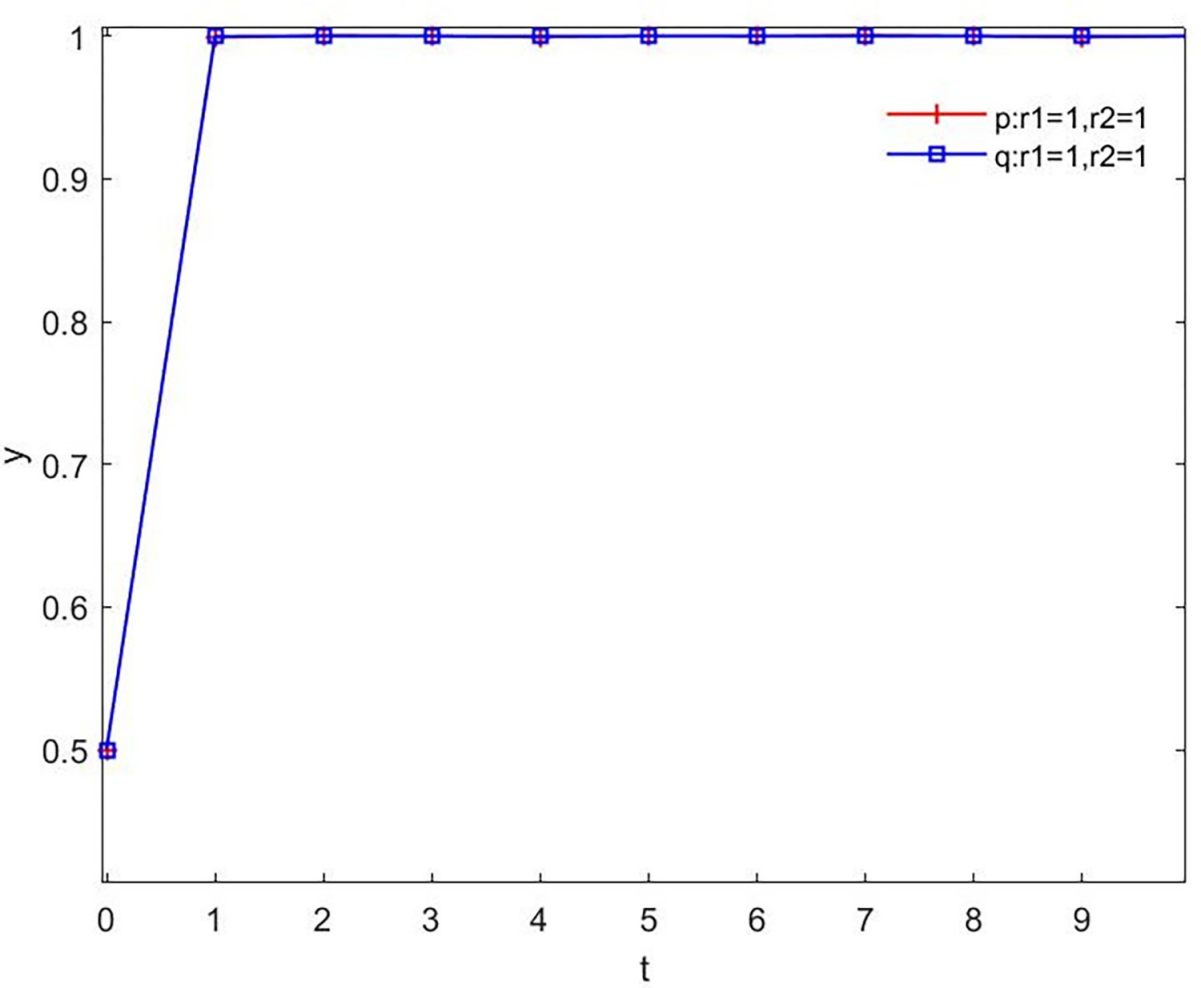
The evolving when government and people are rational. In this diagram, t represents the time of evolution, and y represents the probability that the player will choose (negotiation, understanding).The horizontal and vertical meanings will be the same in subsequent diagrams.

**Fig 3:**
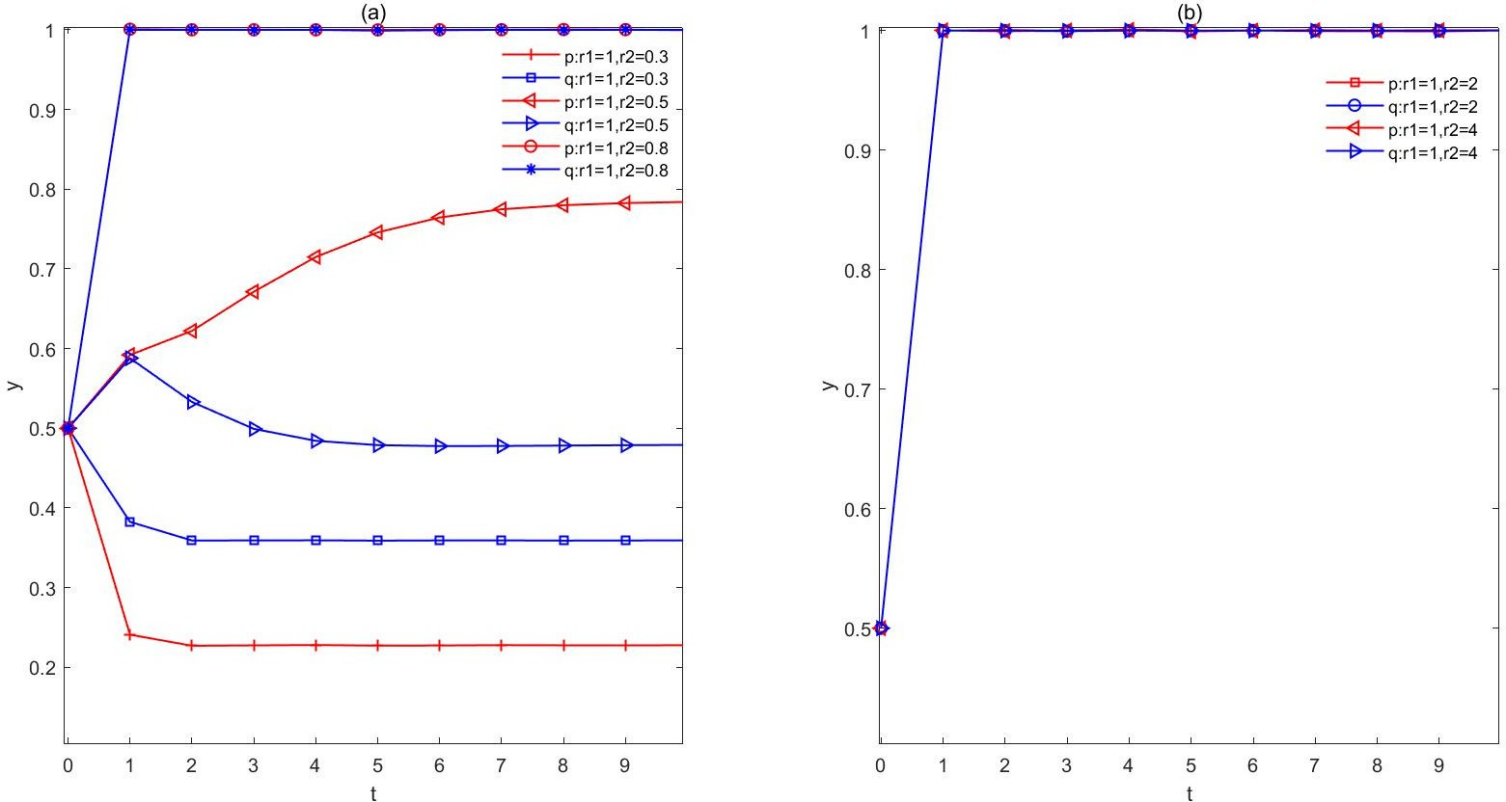
The evolution of rational government. a. The evolution of rational government and pessimistic public. b. The evolution of rational government and optimistic public. Through the setting of the public sentiment index, it can be seen that the evolutionary trajectory has changed significantly. In addition, people’s pessimism had a stronger effect on the results.

(1) Both the government and the public are in a rational state
  When the government and the people are located in a perfectly rational, Fig 2 displays that the probability *p* of the government choosing the negotiation strategy tends to 1, while the probability *q* of the public choosing the understanding strategy approaches to 1. First of all, the government has the initiative in the game, at the beginning of the game to get around the masses cooperating with cognitive and adjust their strategy, makes *p* tend to 1. Then, when people are rational, they also acquire cognition of government negotiation and adjust their own strategies. In the case of comprehensive consideration of gains and losses, the probability *q* converges to 1, that is, they choose the strategy of understanding. But in reality, the underdog often carries emotions that make it hard to be completely rational.

(2) The government is rational and the people are in an emotional state
  In this scenario, we divide two scenarios that distinguish whether people are affected by optimistic or pessimistic emotions for evolutionary analysis. The results are shown in Fig 3. Firstly, the evolutionary situation when the government is rational and the public is pessimistic can be observed in Fig 3(A). When *r*_2_ = 0.8, the probability of the government adopting the negotiation strategy and the public adopting the understanding strategy evolves to 1, indicating that the relatively stable evolutionary strategy(negotiation, understanding) can be obtained when the public is not too pessimistic. As the intensity of public pessimism increases, when *r*_2_ = 0.5 and *r*_2_ = 0.3, the probability of the government adopting a negotiation strategy drops from 0.78 to 0.23. In other words, when *r*_2_ = 0.5, the probability of the government adopting a closed strategy reaches about 0.8. This means that, when considering the intensity of public pessimism, the government is inclined to adopt a closed strategy in the early stage to reduce unnecessary conflicts. At the same time, the probability of people adopting the understanding strategy drops from 0.48 to 0.36. That is to say, with the increase of people’s pessimism, people will increase the probability of choosing the resistance strategy, and under this influence, the probability of the government choosing the closure will also increase.

Secondly, Fig 3(B) shows the evolution when the government is completely rational and the surrounding people are optimistic. When the public’s optimism index *r*_2_ = 2 and *r*_2_ = 4, it can be seen that the probabilities of the government and the public choosing (negotiation, understanding) evolves at a fast speed and stable at 1. This suggests that, when people hold optimistic attitude to the establishment of NIMBY facilities, some people are willing to accept the government’s arrangement and compensation. By this time, in the face of the public’s understanding and support, the government chooses to allow the public to negotiate into the decision, which is less likely to bring social panic effect. In this way, the whole society will be more harmonious and stable, and both the government and the public will reach the relative optimal state of the game.

**Fig 4:**
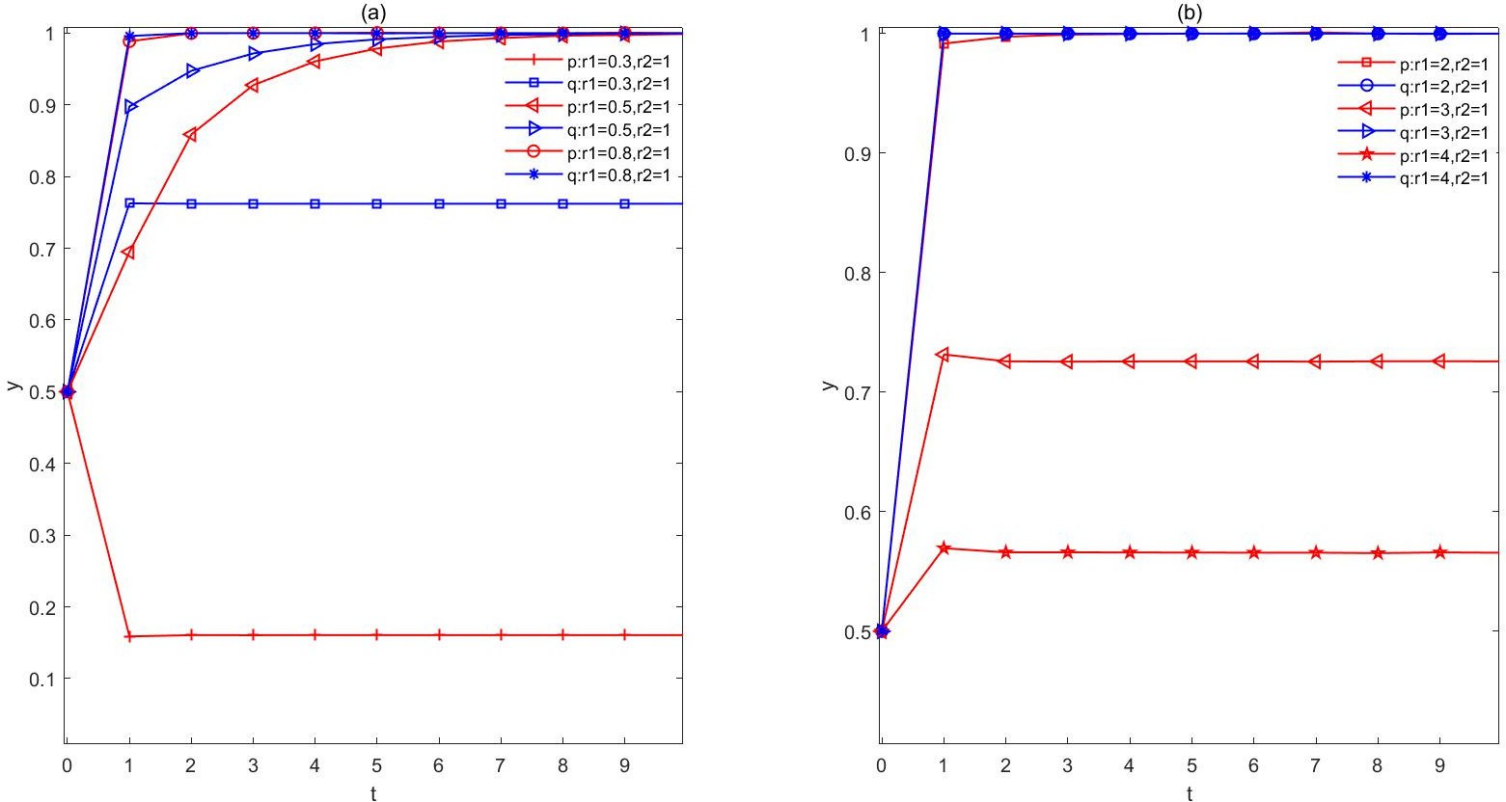
The evolution of rational public. a. The evolution of pessimistic government and rational public. b. The evolution of optimistic government and rational public. By setting a different sentiment index for the government, it can be seen that the evolutionary trajectory has changed significantly. In addition, it is clear that the government’s mood has had a dramatic effect on the results.

(3) The people are rational and the government is in an emotional state
  In this case, we explore the evolution of the trajectory with the different mood of the government, and the results are shown in Fig 4. On the one hand, the evolutionary situation influenced by the pessimistic government is shown in Fig 4(A). When *r*_1_ = 0.8, *r*_1_ = 0.5, and *r*_1_ = 0.3, as the intensity of the government’s pessimism grows, the government will tend to think that negative externalities brought by the construction of NIMBY facilities is large, and will tend to choose a more passive mode of regulation. As a result, the probability that the government will adopt a strategy of closure will rise above 0.8. At the same time, because of the pessimistic plot of the government, the surrounding people will realize some unknown risks brought by the establishment of NIMBY facilities, so the evolution time of choosing to accept the strategy will be correspondingly longer, and the patience of choosing to understand will become less and less, and they will think that the government is weak and incompetent, and realize that opposing the establishment may bring higher profits. Correspondingly, when the government gradually realizes that the public may revolt, it will become more pessimistic and take more negative countermeasures. In this way, the game between the two sides may eventually evolve into a situation of (closure, resistance), where the government becomes inactivity and the society is located in unrest.

On the other hand, when the government holds the optimistic mood, as showed in Fig 4(B), the probability of choice (negotiation, understanding) between the government and the public quickly evolves to 1. That is to say, when the government is in an optimistic state, it will tend to think that the public is willing to understand the establishment of NIMBY facilities, and then it will actively offer substantial compensation to the public and allow the public to negotiate in the decision-making. Likewise, people in a rational state will rationally weigh their individual pros and cons, and tend to choose to accept the policy compensation offered by the government, and support decisions. The two sides evolve into a (negotiation, understanding) of this relatively optimal game strategy. With the increase of the government’s optimism index, the government is less inclined to opt for a negotiating strategy. The main reason for this is that the government actively believes in the rationality of the people, so it does not care much about its own credibility and tends to get more economic benefits. In addition, through the above analysis, it can be obviously observed that the government’s emotions have a greater impact on the results of the evolution of both parties, which also indicates that the government’s sanity plays a significant role in solving NIMBY conflicts.

**Fig 5:**
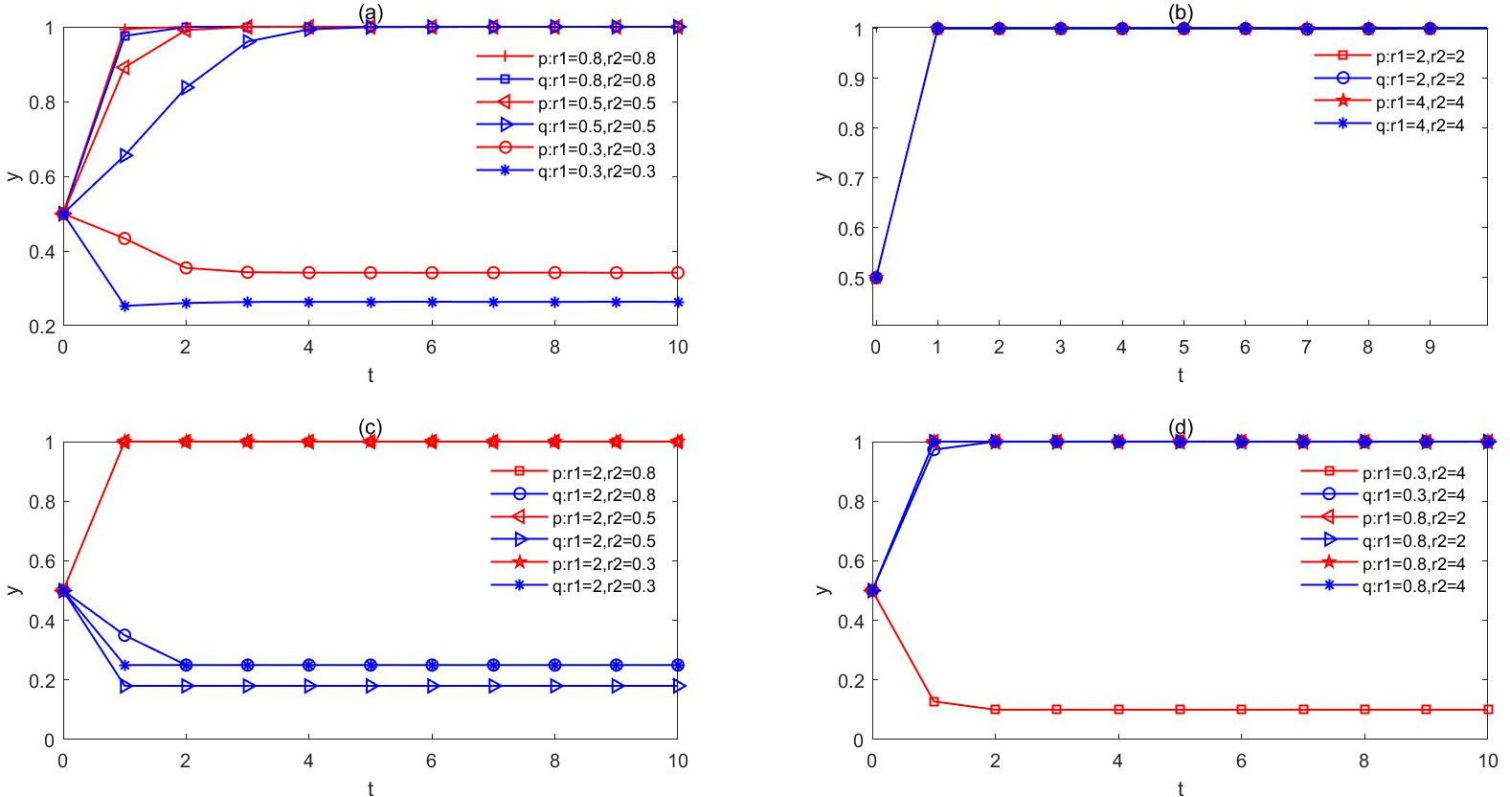
The evolution of different emotional combinations. a. Government pessimism and public pessimism. b. Government optimism and public optimism. c. Government optimism and public pessimism. d. Government pessimism and public optimism. Through the evolutionary analysis of different emotional combinations, it can be found that the emotions of the government and the public have a significant impact on decision-making. Moreover, the mood of the government matters more than the people.

(4) Both the government and citizens have emotional influences
  Considering the different combinations of emotions, this paper simulates and evolves four situations: government pessimism and public pessimism; government optimism and public optimism; government optimism and public pessimism; government pessimism and public optimism. The evolutionary results are presented in Fig 5.

Firstly, when the government and people are pessimistic, as shown in Fig 5(A), clearly the government’s pessimism more severe impact on the strategy of evolution. As the government becomes passive, the public will become more discontent, and the contradiction between the government and the public will increase with the rising of the pessimistic sentiment index of the government, which may eventually lead to the outbreak of NIMBY conflicts, and may even evolve into violent conflicts. Secondly, when both the government and the public are optimistic, Fig 5(B) lists that the probability of the public choosing to the strategy of understanding will soon approach 1. That means, the public will be more willing to understand the government’s policy faster with the increase of the public optimism index. Similarly, when the government sees the public’s cooperation with its work, it tends to increase the public’s participation in decision-making, so as to better safeguard the public’s right to know, to supervise and other rights.

Moreover, Fig 5(C) and 5(D) show the evolutionary trend of different sentiments held by the government and the public. The government tends to quickly choose the strategy of negotiation in the optimistic state, while the public is easily guided by the sentiment when it is overshadowed by the pessimistic mood. They are inclined to believe that the government does not consider them but only to pursue its own interests and achievements, so they choose to oppose the establishment of NIMBY facilities by the government. However, when the government’s pessimism index is not large and the public is optimistic, the two sides will reach a relatively optimal state of the game. Furthermore, the combination of different emotions has a profound effect on the results of evolution, and the mood of the government matters more than the people.

## 5. Conclusions

In this paper, according to the RDEU theory, considering the both sides of the emotional factors and establish the evolutionary game model, and then through the numerical analysis to study the change law of the research results show that emotions can affect both sides behavior strategy at the same time, different kinds of emotions and emotional intensity can lead to a different game result, and get the following conclusions:

1. Under the action of mood, the surrounding people and the government of optimism can bring to solve good results from the conflicts, but the public’s pessimism will not necessarily lead to bad results, depending on the emotional strength compared to the size of the public mood, the government’s emotional state and its intensity size more influence the result of the game, obviously, the government keep rational for adjacent avoid conflicts management work is more important.
2. The emotional intensity of the two main players in the game will accelerate the evolution speed of the strategy. With the increase of the emotional intensity, the faster the consensus of the group strategy is formed, which means that the government needs to do a good job of emotional guidance in order to effectively avoid the occurrence of public revolt.

Depending on the analytical conclusions of the evolutionary game model in this paper, the following suggestions are provided for the current conflicts governance of NIMBY incidents in China:

1. When dealing with the NIMBY conflicts, the government should remain rational, conduct field visits in advance and in-depth visits to the public to enhance the perception of the public’s emotions and make a success of emotional guidance. On the one hand, the government can take to increase subsidies and other welfare policy to improve public awareness and the value of the adjacent from the project, strengthen the construction of the corresponding coordination mechanism, and guide the people involved in the decision-making of science, which can improve the public to the government behavior in the emotional identity, so that people with an optimistic mood to treat NIMBY facilities construction problems. On the other hand, the government can scientifically publicize the NIMBY project by means of slogans, brochures and lectures, thus reduce the risk perception bias of the public and reduce the public’s excessive cognition of the negative externalities of NIMBY facilities. During the actual construction and operation of NIMBY facilities, the government should protect the public’s right to know and right to supervise, so as to decrease the public’s anxiety and pessimism.
2. In NIMBY conflicts, with the improvement of public pessimism intensity, people would be more inclined to put interpretation NIMBY problems as a moral and ethical justice rather than scientific problems, people tend to think of themselves as victims of government policy, it will lead to irrational destructive collective protests, great damage to the country’s social and public itself. Therefore, people need to improve the ability of its own rational and reasonable assessment of risk avoidance, through formal channels to express public opinion and demand, maintain the social and collective public interest, have confidence in the local government departments concerned, and participate scientifically in decision-making. This is not only beneficial for the government to keep the rational, also helps to promote the decision-making of NIMBY facilities to be mature and rational.

This research also has demerits and limitations. Due to the sensitivity of the NIMBY conflicts topic, a great many data and information will not be available to query through official channels, also could not be reached through related people further understand the conflicts, therefore, this paper only carries out simulation analysis based on the information that can be queried and reasonable hypothesis data, hoping to provide ideas for the future management of NIMBY conflicts. Finally, it is hoped that future scholars can conduct more in-depth research and analysis on the basis of existing NIMBY governance theories from more perspectives. It is estimated that with the improvement of government governance ability in the future, the governance of NIMBY conflicts will be more standardized.

## References

[1] Han ZY, Weng WG, Yang LX. The scientific background, goal and organization management of the major research project "unconventional emergency management research". Science Foundation of China. 2009; 2(4):215–220. 10.16262/j.cnki.1000-8217.2009.04.008.

[2] Esaiasson P. NIMBYism-a re-examination of the Phenomenon. Social Science Research. 2014; 8:185–195. doi: 10.1016/j.ssresearch.2014.06.005 25131284

[3] DeWall CN, Baumeister RF, Chester DS, Bushman BJ. How Often Does Currently Felt Emotion Predict Social Behavior and Judgment? A Meta-Analytic Test of Two Theories. Emotion Review. 2016; 8(2):136–143. 10.1177/1754073915572690.

[4] Lang G, Xu Y. Anti-incinerator campaigns and the evolution of protest politics in China. Environmental Politics. 2013; 22(5):832–848. 10.1080/09644016.2013.765684.

[5] Sun LL, Yung EHK, Chan EHW, Zhu DJ. Issues of NIMBY conflict management from the perspective of stakeholders: A case study in Shanghai. Habitat International. 2016; 53:133–141. 10.1016/j.habitatint.2015.11.013.

[6] Kikuchi R, Gerardo R. More than a decade of conflicts between hazardous waste management and public resistance: A case study of NIMBY syndrome in Souselas (Portugal). Hazard Mater. 2009;172:1681–1685. 10.1016/j.jhazmat.2009.07.062. 19679393

[7] Soltani A, Hewage K, Reza B, Sadiq R. Multiple stakeholders in multi-criteria decision-making in the context of Municipal Solid Waste Management: A review. Waste Management. 2015; 35:318–328. doi: 10.1016/j.wasman.2014.09.010 25301545

[8] Sullivan AM. Siting Noxious Facilities—A Siting Lottery With Victim Compensation. Journal of Urban Economics. 1992;31(3):360–374. 10.1016/0094-1190(92)90062-P.

[9] Revelle C, Cohon J, Shobry D. Simultaneous Siting And Routing in The Disposal of Hazardous Wastes. Transportation Science. 1991; 25(2):138–145. 10.1287/trsc.25.2.138.

[10] Zattoni A. Who Should Control a Corporation? Toward a Contingency Stakeholder Model for Allocating Ownership Rights. Journal of Business Ethics. 2011; 103:255–274. 10.1007/s10551-011-0864-3.

[11] Lee MKK. Effective Green Alliances: An Analysis of How Environmental Nongovernmental Organizations Affect Corporate Sustainability Programs. Corporate Social Responsibility and Environmental Management. 2019; 26(1):227–237. 10.1002/csr.1674.

[12] Cong XH, Wang L, Ma L, Skibnewski M. Exploring Critical Influencing Factors for the Site Selection Failure of Waste-to-energy Projects in China Caused by the "Not in My Back Yard" Effect. Engineering Construction and Architectural Management. 2020;28(6):1561–1592. 10.1108/ECAM-12-2019-0709.

[13] Byung DS, James RM, Young DK. Efficient location and allocation strategies for undesirable facilities considering their fundamental properties. Computers&Industrial Engineering. 2013; 65(3):475–484. 10.1016/j.cie.2013.03.009.

[14] Anna D, Edward C, Jens N. Public participation and local environmental planning Testing factors influencing decision quality and implementation in four case studies from Germany. Land use policy. 2015; 46:211–222. 10.1016/j.landusepol.2015.02.010.

[15] O’hare M. Not On My Block You Don’t: Facility Siting and the Strategic Importance of Compensation. Public Policy. 1977; 25(4):407–458. 10.2172/5221104.

[16] Takahashi LM. Contraversial facility siting in the urban environment resident and planner perceptions in the United States. Environment and Behavior March. 1998; 30(2):184–215. 10.1177/0013916598302004.

[17] Cowan S. NIMBY syndrome and public consultant policy: The implications of a discourse analysis of local responses to the establishment of a community mental health facility. Health and Social Care in the Community. 2003;11(5):379–386. doi: 10.1046/j.1365-2524.2003.00439.x 14498834

[18] Teo MM, Loosemore M. Understanding Community Protest from A Project Management Perspective: A Relationship-based Approach. International Journal of Project Management. 2017; 35(8):1444–1458. 10.1016/j.ijproman.2017.08.004.

[19] Zhang L, Tong X. Social Generation Mechanism of "NIMBY" Action. Journal of Jiangsu Institute of Administration. 2013; (01):64–70. 10.3969/j.issn.1009-8860.2013.01.010.

[20] Terri M, Michele R, Silvia R. The false consensus effect: A trigger of radicalization in locally unwanted land uses conflicts? Journal of Environmental Psychology, 2015; 42:76–81. 10.1016/j.jenvp.2015.03.001.

[21] Ma L, Cong XH. Social Stability Risk Assessment of NIMBY Major Projects by OWA, Matter-element, and Cloud Model. Journal of Intelligent & Fuzzy Systems. 2019; 36(3):2545–2556. 10.3233/JIFS-181259.

[22] Devine-Wright P. Public engagement with large—scale renewable energy technologies: breakingthe cycle of NIMBYism. Wiley Interdisciplinary Reviews: Climate Change. 2011; 2(1):19–26. 10.1002/wcc.89.

[23] Hu XM. Risk Events of Social Stability in Sensitive Engineering—Process Model and Action Logic of Participants. Journal of the Chinese Academy of Governance. 2016; (02):58–62. 10.14063/j.cnki.1008-9314.2016.02.033.

[24] Madariaga A, Allain M. Contingent Coalitions in Environmental Policymaking: How Civil Society Organizations Influenced the Chilean Renewable Energy Boom. Policy Studios Journal. 2020; 48(3):672–699. 10.1111/psj.12298.

[25] Yi G, Yang GS. Research on the Tripartite Evolutionary Game of Public Participation in the Facility Location of Hazardous Materials Logistics from the Perspective of NIMBY Events. Sustainable Cities and Society. 2021; 72(5):103017. 10.1016/j.scs.2021.103017.

[26] He XL, Yang XF. Research Progress of Environmental NIMBY Effect and Its Governance Mechanism from the Multi-disciplinary Perspective. Urban Development Studies. 2020; 27(10):28–33. 10.3969/j.issn.1006-3862.2020.10.005.

[27] Wang Y, Li H, Zuo J, Wang Z. Evolution of online public opinions on social impact induced by NIMBY facility. Environmental Impact Assessment Review. 2019; 78:106290. 10.1016/j.eiar.2019.106290.

[28] Wang Y, Shen C, Bartsch K, Zuo J. Exploring the trade-off between benefit and risk perception of NIMBY facility: A social cognitive theory model. Environmental Impact Assessment Review. 2021; 87:106555. 10.1016/j.eiar.2021.106555.

[29] He L, Yang Q, Liu XX, Fu LM, Wang JM. Exploring Factors Influencing Scenarios Evolution of Waste NIMBY Crisis: Analysis of Typical Cases in China. International Journal of Environmental Research and Public Health. 2021; 18(4). doi: 10.3390/ijerph18042006 33669669 PMC7922245

[30] Elaine H, John T, Cacioppo RL. Emotional Contagion. Current Directions in Psychological Science. 1993; 2(3). 10.1111/1467-8721.ep10770953.

[31] Hunter S, Leyden K. Beyond NIMBY: Explaining Opposition to Hazardous Waste Facilities. 1995; 23(4):601–619. 10.1111/j.1541-0072.1995.tb00537.x.

[32] Jennifer SL, Dacher K. Beyond valence: Toward a model of emotion-specific influences on judgement and choice. Cognition & Emotion. 2000; 14(4):473–493. 10.1080/026999300402763.

[33] Berkowitz SL. Risk management in a hostile environment.Health plan. 2000; 41(3):89. 10.1049/ep.1980.0189. 11066254

[34] Jenkins J. Strategy of tension: The Belgian terrorist crisis 1982–1986. Taylor & Francis Group. 1990; 13(4–5):299–309. 10.1080/10576109008435838.

[35] Joshua KD, Kazimierz M, Slomczynski I, Tomescu D. Effects of Democracy and Inequality on Soft Political Protest in Europe: Exploring the European Social Survey Data. International Journal of Sociology. 2008; 38(3):36–51. 10.2753/IJS0020-7659380302.

[36] Wang R, Groot G. Who Represents? Xi Jinping’s Grand United Front Work, Legitimation, Participation and Consultative Democracy. Journal of Contemporary China. 2018; 27(112):569–583. 10.1080/10670564.2018.1433573.

[37] Zhang X, Xu JG, Ju Y. Public participation in NIMBY risk mitigation: A discourse zoning approach in the Chinese context. Land Use Policy.2018; 77:559–575. 10.1016/j.landusepol.2018.04.041.

[38] Evensen D, Stedman R. Scale matters: variation in perceptions of shale gas development across national, state,and local levels. Energy Research & Social Science. 2016; 20:14–21. 10.1016/j.erss.2016.06.010.

[39] Huang C, Hu B, Jiang G, Yang R. Modeling of agent-based complex network under cyber-violence. Physica A Statistical Mechanics & Its Applications. 2016; 458:399–411. doi: 10.1016/j.physa.2016.03.066

[40] Junod AN, Jacquet JB, Fernando F, Flage L. Life in the Goldilocks Zone: Perceptions of Place Disruption on the Periphery of the Bakken Shale. Society & Natural Resources. 2018; 31(2):200–217. 10.1080/08941920.2017.1376138.

[41] Boudet HS. Public perceptions of and responses to new energy technologies. Nature Energy. 2019; 4(6):446–455. 10.1038/s41560-019-0399-x.

[42] Chen T, Wang Y, Yang J, Cong G. Modeling public opinion reversal process with the considerations of external intervention information and individual internal characteristics. Healthcare. 2020; 8(2):160. doi: 10.3390/healthcare8020160 32517050 PMC7349120

[43] Guo B, Li KJ. Psychosocial Pathways of Collective Action Participation in NIMBY Conflict: A Regulated Double Mediation Model. International Journal of Electrical Engineering Education. 2020:002072092093142. 10.1177/0020720920931426.

[44] Gong RT. Analysis of Equilibrium Solution of Hawk-dove Game Based on Rank-dependent Expected Utility Theory. Journal of Management Science. 2012; 15(9):35–45. 10.3969/j.issn.1007-9807.2012.09.004.

[45] Xiong GQ, Zhang T, Wang HT. The RDEU Game Model Analysis of Group conflicts under the Influence of Emotion. Chinese Journal of Management Science. 2015; 23(9):162–170. 10.16381/j.cnki.issn1003-207x.2015.09.020.

[46] Tao P, Tong X. NIMBY group incidents and their governance. Nanjing Social Sciences. 2010; (8):63–68. 10.15937/j.cnki.issn1001-8263.2010.08.010.

[47] Zheng JJ, Yu LK, Ma G, Mi HX, Jiao YY. Residents’ Acceptance Towards Waste-to-energy Facilities: Formation, Diffusion and Policy Implications. Journal of Cleaner Production. 2020; 287(9–10):125560. 10.1016/j.jclepro.2020.125560.

[48] Quiggin JA. Theory of Anticipated Utility. Journal of Economic Behavior and Organization. 1982; 3:323–343. 10.1016/0167-2681(82)90008-7.

[49] Diecidue E, Wakker PP. On the intuition of rank-dependent utility. The journal of Risk and Uncertainty. 2001; 23(3):281–298. 10.1023/a:1011877808366.

[50] Neumann JV, Morgenstern O. Theory of games and economic behavior. Princeton, NJ: Princeton University Press; 1944. ISBN: 9780691130613 10.1515/9781400829460.

[51] Nash JF. Equilibrium points in N-person games. Proceedings of the National Academy of Sciences. 1950; 36:48–9. doi: 10.1073/pnas.36.1.48 16588946 PMC1063129

[52] Maynard SJ. Evolution and the theory of games. Cambridge, UK: Cambridge University Press; 1982. ISBN: 9780521288842 10.1017/CBO9780511806292.

[53] Hofbauer J, Sigmund K. Evolutionary games and population dynamics. Cambridge, UK: Cambridge University Press; 1998. ISBN: 9780521625708 10.1017/CBO9781139173179.

[54] Weibull JW. Evolutionary game theory. Cambridge, MA: MIT Press; 1995. ISBN: 9780262731218 10.1007/978-94-015-8516-3_11.

